# Structural studies promote vaccine development: lessons from African swine fever virus

**DOI:** 10.1093/procel/pwaf055

**Published:** 2025-06-27

**Authors:** Yuxia Zhang, Ling Zhu

**Affiliations:** Changping Laboratory, Beijing 102206, China; Key Laboratory of Biomacromolecules (CAS), National Laboratory of Biomacromolecules, CAS Center for Excellence in Biomacromolecules, Institute of Biophysics, Chinese Academy of Sciences, Beijing 100101, China

African swine fever virus (ASFV) is a large, double-stranded DNA virus classified within the family *Asfarviridae*, a clinically notable outlier in Baltimore Group I. The virus harbors a linear double-stranded DNA genome of 170–194 kb, encoding over 150 open reading frames (ORFs), and features a highly complex multi-layered architecture, wreaking havoc on global swine populations and having caused massive culling and economic losses across Asia, Europe, and Africa (Costard et al., 2009; Penrith et al., 2013). The ability of ASFV to evade host immunity, persist in multiple wild *Suidae* reservoirs, including wild boars (*Sus scrofa*), warthogs (*Phacochoerus spp.*), and bush pigs (*Potamochoerus spp.*). Moreover, its resistance to inactivation in contaminated meat products further facilitates long-distance transmission and contributes to the virus’s geographic expansion across both endemic and non-endemic regions (Costard et al., 2009). Unlike other veterinary viral diseases, ASFV remains largely intractable—there is no approved vaccine or antiviral treatment available to date (Urbano and Ferreira, 2022; Zhang et al., 2024). Numbers of vaccine approaches, including using vaccines with naturally or experimentally deleted genes, subunit vaccines based on recombinant proteins, and DNA vaccines (Arias et al., 2017; Gaudreault and Richt, 2019) (Fig. 1A and 1B), have been explored with varying success, often limited by safety concerns or inadequate protection (Bosch-Camós et al., 2020).

**Figure 1.**
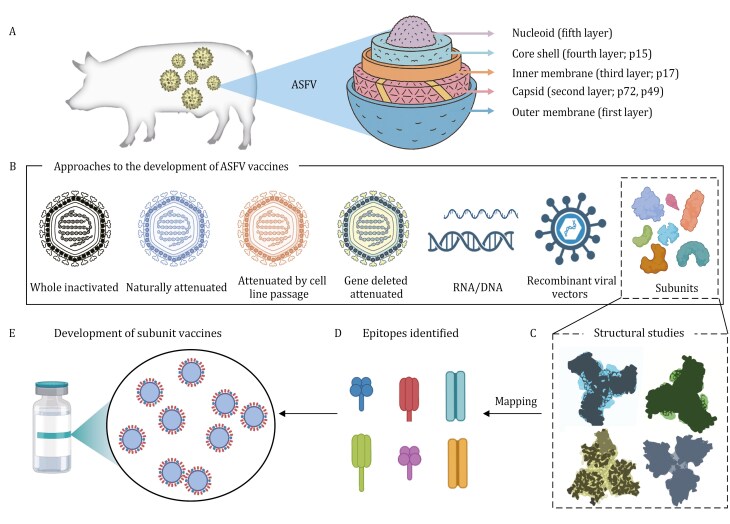
**Multilayered structure of ASFV and emerging strategies for structure-based vaccine design.** (A) Schematic of the multi-layer African swine fever virus (ASFV) structure and representative proteins in each layer. (B) Current approaches to ASFV vaccines development, including whole virus inactive vaccine, naturally attenuated vaccine, attenuated by cell passage vaccine, gene deletion vaccine, nucleic acid vaccine, recombinant viral vector vaccine, and subunit vaccine. (C) Mapping of conformational epitopes on the p72 trimer enables the design of next-generation subunit vaccines. (D and E) Structure-guided approaches include development of epitope-focused synthetic peptides. Additional antigenic targets such as p15/p17 may complement capsid-based strategies to improve protective breadth.

Amid the ongoing crisis, several fundamental barriers continue to obstruct the development of effective interventions against ASFV, including the lack of identification of protective antigens, incomplete understanding of virus–host cell interactions, and limited knowledge relative to the diversity of viral strains currently circulating in natural reservoirs (Rock, 2017; Teklue et al., 2020; Zhu, 2022), which collectively constitute a major global challenge impeding the development of effective countermeasures. These gaps have significantly hindered the design of safe and broadly protective vaccines. Notably, as with many large DNA viruses, the functional properties of ASFV are intimately linked to its complex structural organization. However, a major bottleneck has been the absence of high-resolution structural information that could clarify how the virus assembles and presents antigens to the host immune system. In this context, the work by Wang et al., published in *Science* (2019) (Wang et al., 2019), stands as a pivotal contribution—by revealing the three-dimensional architecture of ASFV (HLJ-2018 strain) at near-atomic resolution, they provide the long-missing foundation upon which rational vaccine and antiviral strategies may now be built.

## Structural breakthroughs and assembly insights of ASFV

Using cryo-electron microscopy (cryo-EM), Wang et al. achieved the reconstruction of the ASFV virion at 4.1 Å resolution, a remarkable feat given the virus’s size (∼260 nm) and multi-layered morphology (Fig. 1A).

This study represents the first near-atomic visualization of the ASFV capsid, revealing both overall symmetry and local complexity (Wang et al., 2019). Furthermore, the authors identified five concentric layers, including the outer membrane (first layer), icosahedral capsid (second layer), an internal membrane (third layer), and a core shell (fourth layer) enclosing the nucleoid (fifth layer) (Fig. 1A). One of the most striking findings in this study is the major capsid protein, p72, was shown to form trimeric units organized into a *T* = 277 icosahedral lattice—far more geometrically complex than the canonical *T* = 16 architecture found in many icosahedral viruses (Wang et al., 2018; Yuan et al., 2018). This high triangulation number reflects the size of the virion and necessitates an intricate network of accessory proteins for structural integrity. Besides, the other four minor proteins M1249L, p17, p49, and H240R were also observed in the capsid layer, performing unique architectural roles to organize into pentasymmetrons and trisymmetrons together with p72 (Wang et al., 2019). Among them, M1249L is especially notable: it forms elongated filaments that interconnect capsomers across long distances (~100 nm), effectively reinforcing the capsid’s curvature and stability. This scaffolding system is reminiscent of structural roles seen in mimiviruses, placing ASFV within the broader context of nucleocytoplasmic large DNA viruses (NCLDVs) (Krupovic and Koonin, 2015; Liu et al., 2019), yet with distinct adaptations.

Moreover, the study maps out protein–protein interaction networks across capsid vertices, offering mechanistic clues into how such a massive particle assembles with fidelity, which is a question that has long perplexed virologists. These insights not only satisfy long-standing structural curiosity but directly inform vaccine design, as we now understand how subunits such as p72 are presented in the native virion.

In addition to the structure reported by Wang et al., Liu et al. also determined a high-resolution structure of ASFV using cryo-EM (Liu et al., 2019). Their study similarly reveals an architecture comprising five concentric layers: the outer membrane, icosahedral capsid, inner membrane, core shell, and nucleoid containing the viral genome, and reaffirms p72 as the principal capsid component together with several minor capsid proteins contributing to structural stability. However, the two maps diverge in their depiction of the “zipper region,” a specialized interface between the two trisymmetrons: Liu et al. observed that the pattern of glue proteins in this region differs markedly from those associated with other capsomers in the adjacent symmetric regions, indicating the presence of a distinct minor capsid protein specifically associated with this interfacial zone. This observation suggests regional specialization within the capsid and highlights additional structural complexity that may influence virion assembly or stability. By integrating these complementary datasets, we gain a more nuanced portrait of ASFV morphogenesis and, importantly, an expanded repertoire of structurally conserved and surface-exposed epitopes. Such insights are indispensable for structure-guided antigen selection and the design of broadly protective vaccine candidates.

## Structure-based vaccinology: toward effective subunit and recombinant vaccines

The development of effective vaccines against ASFV has been historically challenged by its complex structure and its ability to evade the host immune system. Early vaccine candidates, including inactivated and live-attenuated viruses, often faced issues related to safety, limited efficacy, or production difficulties (Rock, 2017; Teklue et al., 2020). Subunit vaccines, particularly those targeting the major capsid protein p72, have been a focal point due to its abundance and immunogenicity (Argilaguet et al., 2012; Lacasta et al., 2014). However, these efforts were hindered by a lack of detailed structural information, leading to suboptimal antigen design and inconsistent immune responses.

The advancements in structural virology have significantly deepened our understanding of ASFV, thereby providing a critical framework for structure-based vaccine design (Liu et al., 2019; Wang et al., 2019; Yu et al., 2024, 2025). A key strategy in this approach involves the detailed structural characterization of viral antigens, particularly the identification of specific antigenic epitopes through high-resolution structures of antigen-antibody complexes (Fig. 1C). Such studies enable precise mapping of functional epitopes, distinguishing between immunodominant but non-protective regions and conserved, broadly neutralizing sites. Notably, Yu et al. employed cryo-EM to resolve the antigenic architecture of the major capsid protein p72 in complex with porcine monoclonal antibodies (Yu et al., 2024). Their analysis identified a structurally conserved “supersite” of vulnerability, composed of conformational epitopes spanning adjacent p72 monomers (Yu et al., 2024). The antibodies targeting the newly identified antigenic supersite exhibited varied neutralization capacities, highlighting its potential as a focal point for immunogen design. By revealing the structural basis of broad and potent antibody recognition, this study provides a framework for developing p72-based subunit vaccines capable of eliciting cross-protective immune responses across diverse ASFV genotypes. In addition to p72, other viral proteins are gaining attention as complementary immunogens. The p15 protein in the core-shell layer, for example, has been structurally and immunologically characterized as an accessory antigen (Jia et al., 2017). Recent studies have demonstrated that p15 can induce specific and robust antibody responses, making it a promising candidate for inclusion in multicomponent subunit vaccine formulations (Fu et al., 2020; Yu et al., 2025).

The integration of structural insights into vaccine design strategies holds promise for overcoming previous challenges in ASFV vaccine development. By focusing on leveraging the structural understanding of potential viral neutralizing epitopes of capsid proteins like p30, p54, p72, and p15 (Fig. 1C–E), it is feasible to design subunit vaccines that elicit robust and protective immune responses. Such structure-informed approaches are instrumental in advancing toward effective and safe ASFV vaccines.

## Concluding thoughts: structural insights guiding solutions

Wang et al.’s work marks a turning point in ASFV research. The meticulous cryo-EM reconstruction reveals not only what ASFV looks like, but how it builds itself, stabilizes its massive capsid, and presents antigens. These are not mere descriptive achievements; they redefine what is now possible in the pursuit of vaccines and therapeutics for ASFV.

Looking beyond ASFV, the era of structure-guided vaccinology has repeatedly demonstrated that understanding viral proteins at the atomic level enables the rational design of effective immunogens. A prominent example is respiratory syncytial virus (RSV), where decades of vaccine failures were ultimately overcome by solving the prefusion structure of the F glycoprotein, which is an achievement that directly led to the development of the next-generation RSV vaccines (McLellan et al., 2013; Papi et al., 2023). Similarly, for SARS-CoV-2, early cryo-EM studies of the spike glycoprotein allowed for the engineering of stabilized prefusion conformations, which became the antigenic basis for multiple successful mRNA and recombinant protein vaccines (Cao et al., 2022; Cui et al., 2022; Lv et al., 2020; Wang et al., 2022). In the case of influenza, structural elucidation of hemagglutinin informed the design of broadly neutralizing antibodies and novel chimeric antigens targeting conserved epitopes (Xuan et al., 2011). These cases collectively illustrate how structural virology can move beyond descriptive science to become a foundational tool for rational vaccine development.

The structural dissection of its virion opens the door to vaccine constructs that are not only immunogenic, but properly folded, stable, and manufacturable. With a clear view of the viral particle, vaccine developers are no longer working in the dark.

## Data Availability

Not applicable.
